# Syphillitic Malformation of Teeth

**Published:** 1862-05

**Authors:** 


### SYPHILITIC MALFORMATION OF TEETH.

At a meeting of the Pathological Society of London, the proceedings of which we published in the Medical Times and Gazette, Mr. Barwell exhibited a cast of the teeth from a syphilitic child, upon which Mr. Hutchinson remarked that "he did not believe one tenth of the cases of malformation of the teeth which came under notice had anything whatever to do with syphilis. The malformation which was diagnostic of that disease was a special, very peculiar, and comparatively rare one. It consisted of dwarfing and notching of the central incisors of the upper set. If this condition of the pair of teeth were not present, all other deformities should count for nothing. He had met with no single case tending to shake his confidence in the value of this condition as a reliable symptom. On the contrary, all recent observations had tended to confirm it. It was, however, a condition which varied in degree, and which required some practice in its appreciation. In many cases of hereditary syphilis the teeth were but little malformed, and in some they might even escape altogether. He believed that the malformation was due to inflammation of the dental structures at an early period of infantile life. If a syphilitic infant escaped an attack of stomatitis the teeth would probably escape malformation, just as if, should no inflammation of the nasal passages occur, the nose would escape the deformity, which is usually so marked in those children. He had many opportunities for seeing families of syphilitic children, and had always found the malformation of teeth most marked in the eldest, and becoming gradually less so in the younger members. He would venture to add, as a precaution, that those peculiarities were never met with in the first set of teeth, since many mistakes had come to his knowledge in consequence of inattention to this fact."

